# The Deleterious Effects of COVID-19 in the Peripartum Period: A Case Report

**DOI:** 10.3390/pediatric13020041

**Published:** 2021-06-16

**Authors:** Alixandria F. Pfeiffer, Rosylyn James, Barbara K. Neuhoff, Wilson B. Pfeiffer, David R. Lowery, Syed A. A. Rizvi

**Affiliations:** 1 Department of Obstetrics and Gynecology, University of Texas Health San Antonio, San Antonio, TX 78229, USA; fiorealixandria@gmail.com (A.F.P.); jamesr2@uthscsa.edu (R.J.); neuhoff@uthscsa.edu (B.K.N.); 2 Department of Anesthesia, Brooke Army Medical Center, San Antonio, TX 78229, USA; wilson.pfeiffer@gmail.com (W.B.P.); lowerymd@gmail.com (D.R.L.); 3 Department of Pharmaceutical Sciences, Hampton University School of Pharmacy, Hampton, VA 23669, USA

**Keywords:** COVID-19, pregnancy, co-morbidities, complications, puerperium

## Abstract

While the Coronavirus Disease 2019 (COVID-19) pandemic continues to wreak havoc across the nation and the globe as one of the most significant global health crises of our time, recent attention has been turned to the effects of COVID-19 on pregnancy and the puerperium. Although most cases have been asymptomatic, for some patients, the disease may be accompanied by serious complications such as pneumonia, acute respiratory distress, multi organ failure, and death. Several case studies have noted that patients with co-morbidities are at a significant risk of these complications. In a recent systematic review and meta-analysis, authors conclude that cardiovascular disease was associated with increased composite poor outcome in patients with COVID-19. The following case report highlights the multi-system complications and severity of symptoms that can take place after childbirth in a patient with co-morbid obstetric and prenatal conditions and an initially asymptomatic COVID-19 infection.

## 1. Introduction

It is well known that obstetric patients with co-morbid cardiovascular diseases are at an increased risk of obstetric complications including pre-eclampsia, eclampsia, stroke, and myocardial infarction [1,2,3]. In a recent study of deceased COVID-19 patients, the most prevalent comorbidities found were hypertension (prevalence of 46%), diabetes, cardiovascular disease, liver disease, lung disease, malignancy, cerebrovascular disease, COPD, and asthma. Several cohort studies have demonstrated the detrimental effects of coronavirus on maternal morbidity and mortality in the antenatal period. However, with such a rapidly evolving pandemic, little is known about the impact of the diagnosis of COVID-19 in either the asymptomatic or symptomatic patient in the postpartum period [4]. Our patient exhibited evidence of pre-existing hypertension complicated by superimposed pre-eclampsia with severe features, possible undiagnosed pulmonary hypertension, and pre-gestational diabetes.

In the obstetric patient, diagnosed with pro-inflammatory states such as pre-eclampsia coupled with COVID-19, massive endothelial cell dysfunction and vascular damage can lead to severe obstetric morbidity and mortality. These two disease states share various symptomologies including shortness of breath, neurologic disfunction, and laboratory abnormalities. Our patient had pre-existing systemic hypertension, which put her at an increased risk for the development of superimposed pre-eclampsia [5]. 

We hope this manuscript will serve as a teaching tool as well an example to follow for other healthcare providers when encountering such a complicated case.

## 2. Case Presentation

A 47-year-old, G4P2012 Hispanic patient presented to the labor and delivery unit for her scheduled repeat cesarean section at 37 weeks in the setting of two previous cesarean sections, uncontrolled Type 2 diabetes mellitus with polyhydramnios, chronic hypertension, and morbid obesity. Admission labs (including complete blood count, syphilis screen, and hepatitis B surface antigen) were all within normal limits. The patient’s admission labs showed a hemoglobin of 13.0 g/dL, hematocrit of 39.6%, and platelets of 158,000/mm^3^. Forty-eight hours prior to presentation to the labor and delivery unit, the patient received routine preadmission nucleic acid COVID-19 screening per hospital policy and tested positive. She denied any recent symptoms of COVID-19 infection and remained asymptomatic through her planned delivery. The patient received a combined spinal epidural, which provided adequate anesthesia for the duration of the procedure. The patient’s delivery was complicated by a postpartum hemorrhage (1500 mL estimated blood loss) likely secondary to a hysterotomy extension created to assist in delivery of a 4980 g infant. The patient was admitted to the postpartum unit, and in the setting of morbid obesity and postpartum state, was started on enoxaparin 30 mg BID for deep venous thrombosis prophylaxis. Postoperative labs (drawn approximately 2 h after surgery) showed a hemoglobin of 11.9 g/dL, hematocrit of 36.2%, and a platelet level of 144,000/ mm^3^. On postpartum day two, the patient had severe hypertension with transaminitis and was diagnosed with postpartum superimposed pre-eclampsia with severe features. She was started on a 24-h magnesium sulfate infusion for seizure prophylaxis. The patient’s labs remained stable, with the nadir of her hemoglobin and hematocrit levels occurring on postpartum day three, at 9.8 g/dL and 29.0%, respectively. On postpartum day three, the patient continued to have severe hypertension and was treated with intravenous and oral antihypertensives (Hydralazine 10 mg × 1 followed by scheduled Labetalol 300 mg BID, increased from her home dose of 100 mg BID). Later that day, she developed a fever to 38.3 °C on one isolated temperature check, with benign physical exam findings. Incision, pelvic, and breast examinations as well as other vital signs were within normal limits. On postpartum day four, the patient developed a second fever of 39.11 °C with respiratory desaturations to the high 80 s and a persistent non-productive cough. A sepsis work-up was initiated and the patient was started on oxygen therapy via nasal cannula to maintain oxygen saturations of >92%. A pulmonary embolism was high on the differential diagnosis along with pneumonia and sepsis. Of note, post-operative infection including endometritis or surgical site infection was lower on the differential at this time given lack of focal findings on physical exam (including uterine tenderness, abnormal vaginal discharge, and incisional tenderness or drainage).

A computed tomography (CT) chest with pulmonary embolism protocol was ordered and demonstrated “diffuse bilateral multifocal ground glass opacities and findings consistent with pulmonary hypertension to include a mildly enlarged pulmonary arterial trunk (3.2 cm).” Given concern for development of a COVID-19 pneumonia with a possible superimposed bacterial component, the patient was started on broad spectrum antibiotics, a five-day course of oral steroids, and a five-day course of Remdesivir with oversight by the hospital’s infectious disease team. A transthoracic echocardiogram was obtained, which showed grossly normal chamber size in all four chambers, trace mitral regurgitation and tricuspid regurgitation, left ventricular ejection fraction of approximately 68%, normal right ventricular systolic function, and pulmonary arterial systolic pressure of 55 mmHg with an estimated central venous pressure of 15 mmHg. The patient subsequently developed increased work of breathing despite oxygen therapy and episodes of tachypnea to the 30s and 40s, so the Medical Intensive Care Unit (MICU) team was consulted and provided recommendations for management. She was determined to be stable for close monitoring on labor and delivery and was not transferred to the MICU. Noninvasive positive pressure ventilation (CPAP) was then initiated for nightly use as an adjunctive therapy to attempt to minimize atelectasis and maximize oxygenation. Additionally, due to the concern for a hypervolemic status, furosemide 20 mg daily was started for a total of 5 days. On postpartum day six, the patient was stable and able to maintain oxygen saturations on 1.5 L of oxygen via nasal cannula. On postpartum day seven, the patient was then weaned to room air and demonstrated signs of symptomatic improvement. On postpartum day eight, the patient continued to improve on room air and her furosemide was decreased to 10 mg daily. By postpartum day 10, the patient had completed her steroid taper as well as her antibiotic regimen and was subsequently discharged with close follow-up with the obstetrics team and the rest of her ancillary teams, including Pulmonology and Family Medicine.

Vertical transmission of viruses from mother to the child can transpire in utero, intrapartum, or via breastfeeding; there are several published reports of such events [6]. In our case, the patient’s infant had initially tested negative for COVID-19 at 24 h and 48 h of life but subsequently developed respiratory distress and sepsis and later tested positive for COVID-19. The infant was placed in isolation, clinically stable, and received care per neonatology. Presently, the patient is doing well and is establishing care in the outpatient setting for further optimization of her co-morbidities; her infant recovered well in the Neonatology Intensive Care Unit and was discharged home on day of life 37.

## 3. Discussion

Our patient had findings on both computed tomography and transthoracic echocardiogram consistent with pulmonary hypertension, to include an enlarged pulmonary arterial trunk (3.2 cm) and an increased pulmonary arterial pressure (55 mmHg), which could have been pre-existing or could have been acute onset secondary to COVID-19 and/or pre-eclampsia. Pre-eclampsia is characterized by events that can result in systemic vascular endothelial damage with resultant vasospasm, transudation of plasma, and ischemic/thrombotic sequelae [6]. Emerging data suggest that the expression of the virus in angiotensin-converting enzyme 2 (ACE2) and its downregulation on vascular endothelium and respiratory epithelium can lead to collapse and deterioration of the renin-angiotensin aldosterone system (RAAS) and cardiovascular compromise in the patient [7]. In most women with pre-existing hypertension in pregnancy, superimposed pre-eclampsia commonly develops earlier in pregnancy due to physiologic rises in blood pressure after the early third trimester [6]. Our patient developed superimposition in the postpartum period, likely secondary to additive and/or inciting effects of COVID-19 as described above. Following delivery of the fetus, maternal peripheral resistance rises and left ventricular workload grows, which is further aggravated by mobilization of pathologic interstitial fluid, which can exacerbate both diastolic and systolic function. This combination can lead to pulmonary edema and worsening lung disease, which was also exhibited by our patient [8]. As shown in this case, patients with asymptomatic COVID-19 can quickly decompensate in the postpartum period due to the synergistic effects of natural postpartum physiology and pro-inflammatory viral states.

Furthermore, as our patient experienced, the additional insult of COVID-19 on the delicate balance of pro- and anti-coagulation in pregnancy can exacerbate the pre-existing risk of obstetric hemorrhage. Pregnant and postpartum patients are already at risk of hematologic complications like disseminated intravascular coagulation (DIC) and thrombotic microangiopathies [9,10]. In our case, patient had a peak elevated D-Dimer level of 1308 (ng/mL) during her hospitalization, indicating an increased level of fibrin degradation within the vascular system, possibly contributing to her postpartum hemorrhage at delivery. Based on previous case studies in pregnancy, there is a link between the exacerbation of immunologic factors (physiologic rise of coagulation factors in the third trimester) of pregnancy with the cytokine storm provoked by COVID-19 infection. These compounding insults could then lead to worsening peripartum bleeding and hemorrhage secondary to consumption of coagulation factors that favor the expression of tissue factor thrombomodulin and endothelial adhesion molecules, thereby activating fibrinolysis [9,11,12]. Institutions should therefore plan for possible coagulopathic derangements in any patient who tests positive for the virus who is scheduled or presents for delivery of any mode.

Additionally, of heightened importance in the setting of a COVID-19 positive patient, regardless of symptomology, is transparent, closed-loop communication and planning with the department of anesthesia. As demonstrated by our patient, pregnant patients with COVID-19 infection present a complicated physiologic state with many anesthetic implications that can prove to be detrimental if unanticipated. Patients in the peripartum period have greater oxygen requirements at baseline (increased by 20–50%) due to the increased oxygen demand of the fetus [9]. While the majority of patients do not experience an increase in closing capacity during pregnancy, respiratory diseases such as COVID-19 can lead to atelectasis and increased closing capacity, thus decreasing the alveoli available for gas exchange in a state of increased oxygen demand [9]. The physiologic state of pregnancy places the patient in a restrictive state of lung pathology due to the extrinsic pressure exerted on the diaphragm and lungs by the developing fetus. In the supine position, this restrictive state is further increased. To further complicate the situation, lung infections such as COVID-19 can cause transient states of pulmonary edema due to capillary leakage, leading to further worsening of the restrictive pulmonary pathology.

There are commonly four options utilized for anesthesia in patients undergoing cesarean section: spinal anesthesia, epidural anesthesia, combined spinal-epidural anesthesia, and general anesthesia. Each of these modes of anesthesia has its own various effects on the respiratory physiology of the patient. In our case, combined spinal-epidural anesthesia was utilized (though the epidural was not utilized since the spinal anesthetic dose was sufficient for anesthetic coverage for the duration of the case). When utilizing a spinal anesthetic, one of the most detrimental complications is the development of an inadvertently high spinal. A spinal anesthetic that rises above the level of T4 and into the cervical levels can quickly result in hypotension, cardiovascular collapse, respiratory collapse, and loss of consciousness. While there are various mechanisms responsible for these responses, the most commonly understood mechanisms include loss of sympathetic vasomotor function, blockade of the cardiac accelerator fibers (located from T1-T5), and possible paralysis of the phrenic nerve (located at C3–C5). Apnea can quickly result from a combination of phrenic nerve paralysis and medullary hypoperfusion resulting from severe sustained systemic hypotension [9]. Another adverse effect that can result from spinal anesthesia is the possible blockade of intercostal muscles, which are located from T1-T11. When blockade of intercostal muscles occurs, the normal function of providing assistance with expansion and shrinking of the chest cavity to facilitate breathing is diminished, thus providing a further increased work of breathing for the patient. In a patient such as ours with multiple existing pathologic states (pregnancy, pre-eclampsia, COVID-19), it is crucial to be readily aware of the potential complications that can occur from neuraxial anesthesia. Thus, for high-risk patients, there should always be a consideration for conversion to general endotracheal anesthesia in order to better control the respiratory parameters (such as peak pressures, tidal volume, and positive end expiratory pressure, among others), with the hopes of minimizing atelectasis and maximizing alveolar gas exchange. This is of vital importance not only to the mother, but also to the fetus in the moments leading to delivery. Fortunately for our patient, she remained hemodynamically stable throughout the case and did not require conversion to general anesthesia. Additionally, of critical importance is the proper resuscitation of blood volume, plasma, and platelets. As previously discussed, the pregnant COVID-19 patient is disposed to consumption of coagulation factors, leading to prolonged bleeding times and prevention of adequate hemostasis. In addition to balanced resuscitation of blood products, anti-hemolytic agents such as tranexamic acid should be strongly considered. Ultimately, the combination of pregnancy and COVID-19 can present many anesthetic challenges, making the inter-departmental peripartum communication of utmost importance in this specific cohort of patients. It has not escaped our attention—new reports are coming out recently highlighting the deleterious effects of Covid-19 infection on the human reproduction [13]. Several reports have described the attraction between severe acute respiratory syndrome coronavirus 2 (SARS-CoV-2) and the angiotensin-converting enzyme-2 (ACE-2) receptors [14]. The ACE-2 receptors are also found in both male and female reproductive organs [15,16] and thus can potentially cause infertility and possible sexual transmission of the virus [17].

## 4. Conclusions

As shown in the discussion of this case, the peripartum patient with a diagnosis of COVID-19 exhibits a highly labile and complicated physiologic environment. Special attention should be paid to the intrinsic cardiovascular and respiratory status of these patients, and their pre-existing conditions should be closely investigated. The patient should be carefully assessed in collaboration with the anesthesia department throughout the peripartum period. As highlighted in the American Journal of Obstetrics and Gynecology, patients of Hispanic race, advanced maternal age, and with medical co-morbidities are risk factors for COVID-19 sequelae, all of which our patient matched [18]. Therefore, pregnant patients with risk factors and co-morbidities who are diagnosed with COVID-19 should be counseled extensively regarding risks of extended hospital stay, anesthetic complications, intensive care unit admission, need for mechanical ventilation and ventilatory support, or even death. While it is crucial to study and develop ways to care for pregnant patients infected with COVID-19, the importance of prevention cannot be understated. It is of utmost importance for the obstetrician-gynecologist to encourage the COVID vaccine for pregnant patients and especially those at high-risk, given the risk of rapid decompensation [19,20]. There may also be some fetal benefit to vaccination in addition to protection of the mother. In a recent study published in the Pediatric Journal of the American Medical Association, maternal IgG antibodies to SARS-CoV-2 were transferred across the placenta after asymptomatic as well as symptomatic infection during pregnancy, which could lead to neonatal protection from the disease [21,22,23]. As the COVID-19 pandemic continues to evolve, studies will need to focus on optimal treatments of infected pregnant women, administration and timing of vaccinations, and effects on the fetus and neonate.

## Data Availability

Not applicable to this article.
